# Extracting Pulmonary Nodules and Nodule Characteristics from Radiology Reports of Lung Cancer Screening Patients Using Transformer Models

**DOI:** 10.1007/s41666-024-00166-5

**Published:** 2024-05-17

**Authors:** Shuang Yang, Xi Yang, Tianchen Lyu, James L. Huang, Aokun Chen, Xing He, Dejana Braithwaite, Hiren J. Mehta, Yonghui Wu, Yi Guo, Jiang Bian

**Affiliations:** 1 Department of Health Outcomes and Biomedical Informatics, College of Medicine, University of Florida, Gainesville, FL USA; 2 Department of Pharmaceutical Outcomes and Policy, College of Pharmacy, University of Florida, Gainesville, FL USA; 3 Departments of Surgery and Epidemiology, University of Florida, Gainesville, FL USA; 4 Division of Pulmonary, Critical Care, and Sleep Medicine, College of Medicine, University of Florida, Gainesville, FL USA

**Keywords:** Pulmonary nodule, Nodule characteristics, Natural language processing, Deep learning

## Abstract

Pulmonary nodules and nodule characteristics are important indicators of lung nodule malignancy. However, nodule information is often documented as free text in clinical narratives such as radiology reports in electronic health record systems. Natural language processing (NLP) is the key technology to extract and standardize patient information from radiology reports into structured data elements. This study aimed to develop an NLP system using state-of-the-art transformer models to extract pulmonary nodules and associated nodule characteristics from radiology reports. We identified a cohort of 3080 patients who underwent LDCT at the University of Florida health system and collected their radiology reports. We manually annotated 394 reports as the gold standard. We explored eight pretrained transformer models from three transformer architectures including bidirectional encoder representations from transformers (BERT), robustly optimized BERT approach (RoBERTa), and A Lite BERT (ALBERT), for clinical concept extraction, relation identification, and negation detection. We examined general transformer models pretrained using general English corpora, transformer models fine-tuned using a clinical corpus, and a large clinical transformer model, GatorTron, which was trained from scratch using 90 billion words of clinical text. We compared transformer models with two baseline models including a recurrent neural network implemented using bidirectional long short-term memory with a conditional random fields layer and support vector machines. RoBERTa-mimic achieved the best *F*1-score of 0.9279 for nodule concept and nodule characteristics extraction. ALBERT-base and GatorTron achieved the best *F*1-score of 0.9737 in linking nodule characteristics to pulmonary nodules. Seven out of eight transformers achieved the best *F*1-score of 1.0000 for negation detection. Our end-to-end system achieved an overall *F*1-score of 0.8869. This study demonstrated the advantage of state-of-the-art transformer models for pulmonary nodule information extraction from radiology reports.

**Supplementary Information:**

The online version contains supplementary material available at 10.1007/s41666-024-00166-5.

## Introduction

Lung cancer stands as the primary cause of cancer-related death in the United States (U.S.) [1]. Research from the National Lung Screening Trial (NLST) has revealed that low-dose computed tomography (LDCT) is capable of detecting lung cancer in its early stages and significantly lowering mortality rates among high-risk individuals [2]. Following the NLST study, numerous professional societies and medical associations, such as the U.S. Preventive Services Task Force (USPSTF) and the American Cancer Society, have recommended lung cancer screening (LCS) with LDCT for high-risk individuals [3, 4].

Pulmonary nodules are abnormal cell growth that forms lumps in the lungs. Pulmonary nodules and nodule characteristics, such as nodule size, multiplicity, and density, detected in the LDCT are important indicators of nodule malignancy [3]. The nodule characteristics are critical for the diagnosis and treatment of lung cancer, as well as for conducting epidemiologic and outcome studies of lung cancer. For instance, the Lung Imaging Reporting and Data System (Lung-RADS®), a quality assurance tool developed by the American College of Radiology (ACR) for standardizing lung cancer screening reporting and recommendations, is based on the pulmonary nodule characteristics. On the other hand, nodule information is often documented in free text clinical narratives such as radiology reports in electronic health record (EHR) systems, which is not readily accessible for down streaming studies such as those examining adherence to Lung-RADS recommendations for surveillance, and applications that utilize structured data. Manually identifying pulmonary nodules and nodule characteristics from free text reports is time-consuming and cannot scale up to large-scale studies.

In the past few years, researchers have developed several rule-based natural language processing (NLP) systems to extract pulmonary nodules and nodule characteristics concepts from radiology reports [5–8]. Although these rule-based NLP systems show a good performance of capturing pulmonary nodules and the associated characteristics, they are known to have generalizability issues when applied to new datasets with different documenting patterns and styles. Researchers often have to substantially customize the rules when applying rule-based NLP systems to radiology reports from a different data source [9]. Machine learning-based NLP models have better performance and generalizability than rule-based NLP systems. Particularly, recent studies have demonstrated that deep learning-based NLP approaches outperformed not only rule-based but also traditional machine learning-based NLP models [10–13]. Previous studies explored machine learning-based and deep learning-based approaches for clinical information extraction from radiology reports and showed excellent performance [14–17]. However, there is no deep learning-based NLP system for pulmonary nodules and nodule characteristic extraction from the radiology report.

This study aimed to develop an NLP system using state-of-the-art transformer models to extract pulmonary nodules and associated nodule characteristics from clinical narratives in radiology reports. Our NLP system consisted of three sub-tasks: (1) clinical concept extraction [18]—to identify the mentions of nodules and the nodule characteristics, (2) clinical relation identification [19]—to link nodule characteristics to the corresponding nodule concept, and (3) negation detection [20]—to identify the negation mention of the nodules (e.g., no nodules have been detected). To develop the NLP system, we established a retrospective cohort of patients that underwent lung cancer screening and collected their radiology reports and physician order notes from the University of Florida Health (UF Health) Integrated Data Repository (IDR). We systematically examined eight pretrained transformer models from three transformer architectures including bidirectional encoder representations from transformers (BERT) [10], robustly optimized BERT approach (RoBERTa) [21], and A Lite BERT (ALBERT) [22] for clinical concept extraction, relation identification, and negation detection. We compared our transformer models with a recurrent neural network (RNN) implemented using a bidirectional long short-term memory (LSTM) architecture with a conditional random fields layer (BiLSTM-CRFs)—as a baseline model for concept extraction and support vector machines (SVMs) model as a baseline model for relation identification.

## Materials and Methods

### Datasets

#### Data Source

This study used clinical text from the University of Florida (UF) Health integrated data repository (IDR), a clinical data repository that consolidates EHR data from various UF Health clinical and administrative systems. We identified 3080 patients who underwent an LCS between 2012 and 2020 using the Healthcare Common Procedure Coding System (HCPCS) code G0297. Based on this patient cohort, we pulled a total of 120,465 clinical narratives, of which 3771 were radiology reports that documented pulmonary nodule characteristics information. We recruited annotators and randomly selected 400 reports for annotation. This research was approved by the UF Institutional Review Board (IRB201901754).

#### Annotation

We developed initial annotation guidelines based on the nodule information defined in Lung-RADS and iteratively optimized the guidelines in multirounds of annotations. Two annotators (SY and TL) manually identified all pulmonary nodules and their associated characteristics [23]. The final annotation guidelines defined seven categories of nodule concepts and six types of relations between the nodule and characteristics of the nodule. For example, in the sentence “nodule located at the lower lobe,” there is a nodule (‘nodule’) and a ‘site’ characteristic (‘lower lobe’) linked by a nodule-site relation. The relations were annotated at the document level which may cross multiple sentences. If there was a negation attached to a nodule concept such as “No pulmonary nodule,” we annotated a negation attribute to the nodule concept. While performing annotation, we excluded content from general suggestions or references to clinical guidelines that were not directly linked to a particular patient (e.g., “lung nodule follows up algorithm: < 4 mm, CT at 12 months”). We calculated the inter-annotator agreement using Cohen’s kappa [24] from 40 reports annotated by both annotators. Annotation discrepancies were resolved through group discussions of the annotators, NLP experts, and physicians. After annotation, we removed duplicated notes and notes with very few concepts. We randomly divided the annotated notes into a training set and a test set according to a ratio of approximately 8:2. We trained various machine learning models using the training set and evaluated the performance using the holdout test set.

### NLP Methods

**Fig. 1:**
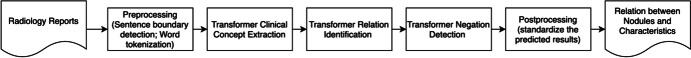
Workflow of our pulmonary nodules extraction system

As shown in Fig. 1, our pulmonary nodules extraction system consists of five modules: preprocessing, concept extraction, relation identification, negation detection, and postprocessing. We employed the preprocessing pipelines established in our prior research (https://github.com/uf-hobi-informatics-lab/NLPreprocessing) [25], which integrated standard NLP procedures including tokenization, text normalization, sentence boundary detection, and data format transformation. Details for each module are provided in Supplement Appendix 1. In the concept extraction module, we adopted state-of-the-art transformer-based NLP models, including BERT, RoBERTa, and ALBERT, and compared them with BiLSTM-CRFs [12] as the baseline. In relation identification, we adopted the same transformer-based NLP architectures mentioned above and compared them with the SVMs as the baseline. In the negation detection module, we approached negation detection as a classification problem, where the transformer models were trained to determine whether a lung cancer nodule mention in clinical text was negated. We explored pretrained transformers from the general English domain (e.g., BERT-base), public available models (e.g., BERT-mimic) pertained on PubMed and Medical Information Mart for Intensive Care III (MIMIC-III) corpora [26], as well clinical specific model pretrained using clinical narrative (e.g., GatorTron). The postprocessing module aggregates results from concept extraction, relation identification, and negation detection into a standard output format. The predicted results are first organized by document id; then, the concept extraction results in BIO format was converted to BRAT format, followed by assigning detected relations to the entities and finally attached the negation results to entities. The amalgamated results are saved into files which allows end-to-end evaluation and results visualization via the BRAT annotation tool. Details of the concept extraction, relation identification, and negation detection modules are described in the following sections.

#### Concept Extraction Module

The goal of the concept extraction module was to extract nodule mentions and nodule characteristics. According to the ACR recommendation, radiologists are required to document nodule characteristics (e.g., size, shape, composition, and margin) of each nodule [3]. We adopted state-of-the-art transformer-based deep learning models for concept extraction. We adopted the standard beginning-inside-outside (BIO) tagging scheme to label the annotated pulmonary nodule and nodule characteristics using BIO tags, where “B” indicates the first token of a concept, “I” indicates tokens inside of a concept, and “O” indicates tokens that do not belong to any concepts. The goal of concept extraction was to classify each token in a sentence into predefined ‘BIO’ categories. The transformer model (e.g., BERT) introduced an algorithm to break words into common sub-tokens to reduce vocabulary size and avoid out-of-vocabulary problems; therefore, a special tag “X” was introduced to label the non-leading sub-token, which is modified from the previous deep learning models (e.g., BiLSTM-CRFs) that rely on word-level ‘BIO’ tags.

We used the BiLSTM-CRFs as the baseline model. The BiLSTM-CRFs is the most adopted deep learning model for concept extraction before transformer-based models. The LSTM component has several “gates” to model the long-distance dependency in language. In this study, we adopted the BiLSTM-CRF architecture by Lample et al. [12]. The model has 2 bidirectional LSTM layers: a word embedding layer and a character embedding layer to transform the input words and characters into vector representations. The last layer is a CRF layer that decodes the hidden states from the word-level bidirectional LSTM to BIO tags and predicts the named entities. We used word embeddings pretrained using the fastText package on de-identified clinical notes from the MIMIC II corpus. The dimension of word embeddings was set to 100.

#### Relation Identification

We approached relation identification as a classification task—to classify a pair of concepts into predefined relation categories. In this study, a relation was defined between a nodule and a characteristic concept. We adopted a two-stage classification procedure developed in our previous study [27], including (1) a binary classifier to determine whether two concepts “has a relation” or “has no relation” and (2) a rule-based procedure to further categorize entity pairs that “has a relation” into the correct relation category based on the entity types. For example, if a candidate entity pair was classified as “has-relation” and one of the entities was nodule and the other was site, then the rule-based procedure would classify it as a “nodule-site” relation. The rule-based procedure in stage 2 worked since there was only one relation category defined between two types of entities. Another challenge in relation identification was to identify the candidate pairs that had relations. Theoretically, we could generate candidate pairs by enumerating combinations among all concepts as there might be a relation in between. However, this would introduce too many negative samples for classification. Instead, we applied the following heuristic rule to reduce combinations: (a) we only kept the concept pairs composed of a nodule entity as the first element and a nodule characteristics entity as the second element and (b) for cross-distance of pairs, we first defined the number of sentence boundaries between the two entities (e.g., 0 for single-sentence relations and 1 for relations across two sentences), then we only considered candidate pairs with cross-distances less than three since we found that, in the training set, 96% of the annotated relations had cross-distances less than three. We also used a unified BERT-based classifier developed by our previous study to handle all candidate pairs with various cross-distances [28, 29].

We used the SVMs as the baseline model. SVMs is widely used in various classification tasks and demonstrated good performance [30] before the emerging of deep learning models. In this study, we used the SVM implementation in the LIBSVM-3.22 package [31] and optimized the regularizer ***C*** and the tolerance of termination criterion ***E***. We used features including the text of entities in candidate pairs and their *n*-grams (*n* = 2,3), *n*-grams (*n* = 2,3,4,5) of context before and after entities in candidate pairs, the token distance between the entities in candidate pairs, and the concept extraction tags of all tokens in the sentences where the candidate pairs are located.

#### Negation Detection

We approached negation detection as a binary classification problem—to classify the observed entities into two predefined categories including “negated” and “non-negated.” We performed the negation detection for each ‘nodule’ entity and then integrated the results with concept extraction and relations in the postprocessing pipeline. For each nodule entity recognized by the concept extraction module, we identified the corresponding sentence, which was fed into transformer models to generate distributed representations from the nodule entity and its contexts. Then, we added a classification layer composed of a linear layer with soft-max activation to calculate a probability score. We experimented with various context window setups, comparing the use of just one sentence to including the sentences before and after as context, and we observed no performance difference. The best performance was achieved when using only the sentence containing the entity and we did not observe improvement by adding nearby sentences.

#### Transformer Models

In this study, we examined eight pertained transformer models from both general English domain and clinical domain. We explored three widely used transformer-based architectures including BERT, RoBERTa, and ALBERT. BERT is a multilayer bidirectional transformer-based encoder model. It was pretrained using masked language modeling (MLM) and next-sentence prediction. RoBERTa has the same architecture as BERT; unlike BERT pertained using static masking pattern generated during preprocessing, RoBERTa is pretrained using a dynamic masked language modeling and optimized using different strategies such as full-sentence without next-sentence prediction (NSP) loss, large mini-batches, and a larger byte-level BPE. (e.g., removing the next-sentence prediction). ALBERT is a simplified version of BERT. It reduces total number of parameters by factorizing the token-embedding layer size, to optimize large-scale configurations and memory efficiency. ALBERT also pretrained using MLM and optimized using sentence-order prediction loss. We examined transformer models pretrained with 110 million parameters, i.e., BERT-base, RoBERTa-base, and ALBERT-base. We also examined their clinical versions fine-tuned using the MIMIC-III corpora, i.e., BERT-mimic, RoBERTa-mimic, and ALBERT-mimic, which were developed in our previous study [25]. We further explored Bio-Clinical BERT [32] and the GatorTron model [33] for comparison. Bio_ClinicalBERT was developed using 0.5 billion words from the MIMIC-III dataset [32] with 110 million parameters. GatorTron was developed utilizing over 90 billion words extracted from de-identified clinical notes from University of Florida (UF) Health, PubMed articles, and Wikipedia, with 345 million parameters, which demonstrated good performance in clinical concept and relation extraction [33].

### Experiment and Evaluation

The baseline BiLSTM-CRFs model was developed using TensorFlow and transformer-based models were developed using PyTorch in our previous work [25, 28]. We chose the best model checkpoints based on the *F*1-scores achieved on the validation set. For transformers, we adopted models pretrained using the MIMIC III corpus (BERT-mimic, RoBERTa-mimic, and ALBERT-mimic) in our previous study [25]. We used the Bio_ClinicalBERT developed by a previous study [32] and used GatorTron model developed in our previous study [33]. For NER, we split the training set further into a sub-training set and a validation set with a ratio of 8:2. We trained the models on the training set and saved the best checkpoints based on the model performance on the validation set. We adopted the early stopping strategy in the training and fixed the training epoch number and batch size as 30 and 4, respectively, for all NER experiments. For relation extraction and negation detection tasks, we adopted a fivefold cross-validation strategy in training set to optimize the model hyperparameters, including the training epoch number (in a range from 3 to 6) and training batch size (4, 8, and 16). We kept all other hyperparameters as default (e.g., learning rate at 1e-5 and random seed at 13) during the experiments. We evaluated NLP models using strict (i.e., the beginning and end boundaries of a concept must be the same with gold standard annotation) micro-averaged precision, recall, and *F*1-score calculated using the official evaluation script from the 2018 n2c2 challenge [34]. To approximate the non-parametric standard deviation for model performances, we adopted the bootstrapping method where we re-generate datasets using different random seeds and repeat the same experiment 20 times and used the obtained results to calculate the standard deviation [35]. The best models were selected according to the cross-validation performances measured as micro-averaged strict *F*1-score. All experiments were conducted using five Nvidia A100 GPUs. The concepts, relations, and negations annotated by human exerts were used as the gold standard for evaluation.

## Results

### Annotation of Pulmonary Nodules and Nodule Characteristics

The two annotators identified a total of 2012 pulmonary nodule concepts and nodule characteristics from 394 notes with an inter-annotator agreement of 0.925. We divided the data into a training, a validation, and a test set. Table 1 shows the distribution of concepts, relations, and negation in the training, validation, and test sets.

**Table 1:** Number of concepts in training, validation, and test set across entity types

Category	Sub-category	Training (*n* = 273 notes)	Validation (*n* = 31 notes)	Test (*n* = 90 notes)
Concept	Nodule	426	43	147
	Site	252	20	93
	Laterality	240	17	92
	Size	221	19	77
	Course	135	5	31
	Texture	111	11	29
	Shape	27	5	11
Relation	Nodule-site	243	19	87
	Nodule-laterality	236	16	87
	Nodule-size	216	19	67
	Nodule-course	135	5	32
	Nodule-texture	116	11	29
	Nodule-shape	27	5	11
Negation	Negated	116	22	24
	Non-negated	288	80	66

### Extraction of Pulmonary Nodules and Nodule Characteristics

Table 2 compares eight transformer-based NLP models with a baseline BiLSTM-CRF model for extracting pulmonary nodule and nodule characteristics. All transformer-based NLP methods outperformed the baseline model in terms of *F*1-score, with the RoBERTa-mimic model achieving the best F1-score of 0.9279 followed by the GatorTron model with a *F*1-score of 0.9274. Noticed that the RoBERTa model achieved a high recall score (0.9747) but a relatively low prevision score (0.8853) while the GatorTron model’s precision and recall scores are more balanced (0.9021 and 0.9542). Supplement Table 1 shows detailed performance of RoBERTa-mimic for the seven categories of pulmonary nodules and nodule characteristics. Among the seven categories, RoBERTa-mimic achieved an excellent *F*1-score for recognizing nodule course (0.9841), and a good *F*1-score for the nodule shape (0.8000).

**Table 2:** Comparison of deep learning models for extraction of pulmonary nodule and nodule characteristics

Model	Precision	Recall	*F*1
BiLSTM-CRFs	0.9066 ± 0.026	0.7688 ± 0.038	0.8320 ± 0.033
BERT-base	0.8868 ± 0.028	0.9697± 0.015	0.9264 ± 0.023
BERT-mimic	0.8750 ± 0.029	0.9722± 0.015	0.9211 ± 0.024
RoBERTa-base	0.8665 ± 0.03	0.9672± 0.016	0.9141 ± 0.025
RoBERTa-mimic	0.8853 ± 0.028	**0.9747± 0.014**	**0.9279 ± 0.023**
ALBERT-base	0.9073 ± 0.026	0.9394± 0.021	0.9231 ± 0.024
ALBERT-mimic	0.8849 ± 0.028	0.9318± 0.022	0.9077 ± 0.026
Bio_ClinicalBERT	**0.9075 ± 0.026**	0.8829± 0.029	0.8950 ± 0.027
GatorTron	0.9021 ± 0.026	0.9542± 0.019	0.9274 ± 0.023

Values in bold indicate the best value per metric. Multiple bold numbers per column signify that there is no statistical difference among the top-performing values for the given metric

### Linking Nodule Characteristics to Nodules

Table 3 compares the eight transformer-based NLP models with the SVMs as the baseline for linking nodule characteristics to pulmonary nodules. All transformer-based NLP methods achieved better *F*1-scores than the baseline model. Both the ALBERT-base model and GatorTron model achieved the best *F*1-score of 0.9737. Supplement Table 2 shows detailed performance of ALBERT-base for six relation categories used to link nodule characteristics to pulmonary nodules. Among the six relation categories, ALBERT-base, which achieved a perfect *F*1-score (1.0000) for linking ‘shape’ to nodules, and an excellent *F*1-score (0.9500) for linking ‘course’ to nodules.

**Table 3:** Comparison of deep learning models to link pulmonary nodule characteristics to pulmonary nodules

Model	Precision	Recall	*F*1
SVMs	0.9372 ± 0.022	0.9731 ± 0.014	0.9548 ± 0.019
BERT-base	0.9338 ± 0.022	0.9732 ± 0.014	0.9531 ± 0.019
BERT-mimic	0.9317 ± 0.022	**0.9923 ± 0.008**	0.9610 ± 0.017
RoBERTa-base	0.9487 ± 0.02	**0.9923 ± 0.008**	0.9700 ± 0.015
RoBERTa-mimic	0.9487 ± 0.02	**0.9923 ± 0.008**	0.9700 ± 0.015
ALBERT-base	**0.9557 ± 0.018**	**0.9923 ± 0.008**	**0.9737 ± 0.014**
ALBERT-mimic	0.9519 ± 0.019	0.9847 ± 0.011	0.9680 ± 0.016
Bio_ClinicalBERT	0.9018 ± 0.027	0.9847 ± 0.011	0.9414 ± 0.021
GatorTron	**0.9557 ± 0.018**	**0.9923 ± 0.008**	**0.9737 ± 0.014**

Values in bold indicate the best value per metric. Multiple bold numbers per column signify that there is no statistical difference among the top-performing values for the given metric

### Negation Detection

Per annotation, there were 162 negated and 454 non-negated nodule entities. Table 4 shows the performance of the eight transformer-based NLP models for negation detection. Two general transformer models (BERT-base and ALBERT-base) and five clinical transformer models (BERT-mimic, RoBERTa-mimic, ALBERT-mimic, Bio_ClinicalBERT, and GatorTron) achieved the best *F*1-score of 1.0000 on the test set. We used the RoBERTa-mimic model in the end-to-end pipeline as it also showed better performance in concept extraction.

**Table 4:** Comparison of deep learning models for negation detection

Model	Precision	Recall	*F*1
BERT-base	1.0000	1.0000	1.0000
BERT-mimic	1.0000	1.0000	1.0000
RoBERTa-base	0.9600 ± 0.017	1.0000	0.9800 ± 0.012
RoBERTa-mimic	1.0000	1.0000	1.0000
ALBERT-base	1.0000	1.0000	1.0000
ALBERT-mimic	1.0000	1.0000	1.0000
Bio_ClinicalBERT	1.0000	1.0000	1.0000
GatorTron	1.0000	1.0000	1.0000

### The End-to-End System

We integrated the best concept extraction model (RoBERTa-mimic), the best relation identification model (ALBERT-base), and the best negation detection model (RoBERTa-mimic) into an end-to-end system. Our end-to-end NLP system for extraction of pulmonary nodule and nodule characteristics achieved an *F*1-score of 0.8869 (precision = 0.8345 and recall = 0.9464).

## Discussion and Conclusion

In this study, we developed an NLP system to extract pulmonary nodules and nodule characteristics from radiology reports. We explored eight state-of-the-art transformer models for concept extraction, relation identification, and negation detection and compared them with two baseline models including BiLSTM-CRFs and SVMs. RoBERTa-mimic achieved the best *F*1-score of 0.9279 for extracting nodule concept and nodule characteristics. ALBERT-base and GatorTron achieved the best *F*1-score of 0.9737 for linking nodule characteristics to pulmonary nodules. Seven out of the eight transformer models achieved the best *F*1-score of 1.000 for negation detection. Our end-to-end system achieved an overall excellent *F*1-score of 0.8869. This study demonstrated the advantage of state-of-the-art transformer models for pulmonary nodule information extraction from radiology reports.

For nodule concepts and nodule characteristics extraction, the clinical transformer RoBERTa-mimic outperformed the other general transformer models pretrained using general English corpora, which is consistent with results from our previous study [25]. Among the lung nodule and characteristic concepts, RoBERTa-mimic had a moderate performance for nodule shape. One potential reason is that the number of nodule shape concepts annotated in the corpus is lower (*N* = 43) than other categories. For relation identification, the ALBERT-base model outperformed clinical transformers pretrained using de-identified clinical notes, indicating that general English context and vocabulary may play an important role in determining the relation types than the medical context. The GatorTron model also achieved the best *F*1-score for relation identification task, this is consistent with previous studies [36]. More future studies are needed to further examine this finding. Seven out of the eight transformer models achieved an *F*1-score of 1.0000 in negation detection, indicating that the negation patterns in radiology reports are very consistent.

Pulmonary nodules and nodule characteristics are important information to determine Lung-RADS scores to categorize radiology findings. NLP systems that can automatically extract pulmonary nodule information from radiology reports are critical to enable medical AI systems leveraging narrative clinical text for lung cancer screening and diagnosis prediction. The NLP system developed in this study is a valuable resource for lung cancer studies that require pulmonary nodules and nodule characteristics from radiology reports. Our NLP system uses the state-of-the-art transformer models and can be adopted for the extraction of other types of nodule information such as that for thyroid nodules.

This study has limitations. First, the nodule characteristics extracted by our NLP system need to be normalized. For example, nodule size can be present in different units of measurement, and the descriptions of shape and texture have many variations. Second, we approached the task of linking nodule characteristics to pulmonary nodules as a relation identification task, future studies need to further explore other potential solutions, such as adopting machine reading comprehension models to identify the characteristics using prompts tuning algorithms [37]. Our future work includes developing NLP pipelines to normalize pulmonary nodules and nodule characteristics and exploring prompt-based solutions for linking nodule characteristics to pulmonary nodules [38].

### Supplementary Information

ESM 1 (DOCX 20 kb)
