# Supplementary material for: Extracting Pulmonary Nodules and Nodule Characteristics from Radiology Reports of Lung Cancer Screening Patients Using Transformer Models
Source: J Healthc Inform Res. 2024 May 17;8(3):463–77. doi: 10.1007/s41666-024-00166-5 (PMC11310180; doi:10.1007/s41666-024-00166-5)
Supplement: Supplementary file 1 — (DOCX 20 kb) [file 41666_2024_166_MOESM1_ESM.docx]

Extracting Pulmonary Nodules and Nodule Characteristics from Radiology Reports of Lung Cancer Screening Patients Using Transformer Models

Shuang Yang^1^, Xi Yang^1^, Tianchen Lyu^1^, James L. Huang^2^, Aokun Chen^1^, Xing He^1^, Dejana Braithwaite^3^, Hiren J. Mehta^4^, Yonghui Wu^1^, Yi Guo^1^, Jiang Bian^1§^

^1^Department of Health Outcomes and Biomedical Informatics, College of Medicine, University of Florida, Gainesville, Florida, USA

^2^Department of Pharmaceutical Outcomes and Policy, College of Pharmacy, University of Florida, Gainesville, Florida, USA

^3^Departments of Surgery and Epidemiology, University of Florida, Gainesville, Florida, USA.

^4^Division of Pulmonary, Critical Care, and Sleep Medicine, College of Medicine, University of Florida, Gainesville, Florida, USA

^§^Corresponding author:

Jiang Bian

bianjiang@ufl.edu.

Department of Health Outcomes and Biomedical Informatics

College of Medicine

University of Florida

Clinical and Translational Research Building, 2004 Mowry Road, PO Box 100177, Gainesville, FL, USA, 32610

**Appendix 1.** Preprocessing modules

The preprocessing module integrated standard NLP procedures including tokenization, text normalization, sentence boundary detection, and data format transformation.

Text tokenization is performed using heuristic rules, including separate punctuation and special symbols (e.g., slash, parenthesis) from words, and fixing concatenations (e.g., missing white space like converting “CancerScreening ” to “Cancer Screening”). This process ensures that the text is broken down into meaningful units for analysis.

Text normalization involves converting text to lowercase; replacing special characters with standardized equivalents, such as symbols like '&' are systematically converted to HTML entities (e.g., '&;'), and whitespace characters such as '\xa0' are replaced with standard spaces; and standardizes the text by converting all content to UTF-8 encoding. These steps streamline the text data, making it consistent and easier to process.

Regarding sentence boundary detection, the script employs a rule-based approach that considers punctuation, special cases, and contextual clues to identify sentence boundaries accurately. Special attention is given to complex cases often found in medical texts, ensuring robust detection even in challenging scenarios.

Since different transformer models adopted different tokenization strategies (eg, WordPiece for BERT, byte pair encoding for RoBERTa, and SentencePiece for XLNet), our preprocessing automatically picked the appropriate tokenizer according to the transformer model in use and aligned the word-level ‘BIO’ tags to the sub token-level ‘BIO’ tags.

**SupplTable 1.** Roberta-mimic performance for extraction of pulmonary nodule and nodule characteristics

| **Entity Type** | **Precision** | **Recall** | **F1** |
| --- | --- | --- | --- |
| Nodule | 0.8387 ± 0.033 | 0.9455 ± 0.020 | 0.8889 ± 0.028 |
| Site | 0.8523 ± 0.032 | 0.9868 ± 0.010 | 0.9146 ± 0.025 |
| Laterality | 0.8659 ± 0.030 | 1.0000 | 0.9281 ± 0.023 |
| Size | 0.9571 ± 0.018 | 1.0000 | 0.9781 ± 0.013 |
| Course | 1.0000 | 0.9688 ± 0.015 | 0.9841 ± 0.011 |
| Texture | 0.9697 ± 0.015 | 0.9697 ± 0.015 | 0.9697 ± 0.015 |
| Shape | 0.7500 ± 0.039 | 0.8571 ± 0.031 | 0.8000 ± 0.036 |

**SupplTable 2.** ALBERT-base performance link pulmonary nodule characteristics to pulmonary nodules.

| **Entity Type** | **Precision** | **Recall** | **F1** |
| --- | --- | --- | --- |
| nodule-size | 0.9655 ± 0.016 | 0.9825 ± 0.012 | 0.9739 ± 0.014 |
| nodule-site | 0.9437 ± 0.021 | 1.0000 | 0.9710 ± 0.015 |
| nodule-shape | 1.0000 | 1.0000 | 1.0000 |
| nodule-course | 0.9143 ± 0.025 | 1.0000 | 0.9552 ± 0.018 |
| nodule-texture | 0.9714 ± 0.015 | 0.9714 ± 0.015 | 0.9714 ± 0.015 |
| nodule-laterality | 0.9692 ± 0.015 | 1.0000 | 0.9844 ± 0.011 |
